# MicroRNA-223 Attenuates Hypoxia-induced Vascular Remodeling by Targeting RhoB/MLC2 in Pulmonary Arterial Smooth Muscle Cells

**DOI:** 10.1038/srep24900

**Published:** 2016-04-28

**Authors:** Yan Zeng, Xiaoying Zhang, Kang Kang, Jidong Chen, Zhiqin Wu, Jinyong Huang, Wenju Lu, Yuqin Chen, Jie Zhang, Zhiwei Wang, Yujia Zhai, Junle Qu, Ramaswamy Ramchandran, J. Usha Raj, Jian Wang, Deming Gou

**Affiliations:** 1 Shenzhen Key Laboratory of Microbial Genetic Engineering, Shenzhen Key Laboratory of Marine Bioresource and Eco-environmental Science, College of Life Sciences, Shenzhen University, Shenzhen, Guangdong 518060, China; 2 Key Laboratory of Optoelectronic Devices and Systems of Ministry of Education and Guangdong Province, College of Optoelectronic Engineering Shenzhen University, Shenzhen, Guangdong 518060, China; 3 Department of Physiology, Shenzhen University Health Science Center, Shenzhen, Guangdong 518060, China; 4 Guangzhou Institute of Respiratory Diseases, State Key Laboratory of Respiratory Diseases, The First Affiliated Hospital, Guangzhou Medical University, 151 Yanjiang Road, Guangzhou, Guangdong 510120, China; 5 Department of Cardiovascular Surgery, Shenzhen Sun Yat-Sen Cardiovascular Hospital, Shenzhen, Guangdong 518060, China; 6 Department of Pediatrics, University of Illinois at Chicago, Chicago, IL 60612, USA; 7 Division of Pulmonary and Critical Care Medicine, Johns Hopkins University School of Medicine, Baltimore, MD 21224, USA

## Abstract

There is growing evidence that microRNAs are implicated in pulmonary arterial hypertension (PAH), but underlying mechanisms remain elusive. Here, we identified that miR-223 was significantly downregulated in chronically hypoxic mouse and rat lungs, as well as in pulmonary artery and pulmonary artery smooth muscle cells (PASMC) exposed to hypoxia. Knockdown of miR-223 increased PASMC proliferation. In contrast, miR-223 overexpression abrogated cell proliferation, migration and stress fiber formation. Administering miR-223 agomir *in vivo* antagonized hypoxia-induced increase in pulmonary artery pressure and distal arteriole muscularization. RhoB, which was increased by hypoxia, was identified as one of the targets of miR-223. Overexpressed miR-223 suppressed RhoB and inhibited the consequent phosphorylation of myosin phosphatase target subunit (MYPT1) and the expression of myosin light chain of myosin II (MLC2), which was identified as another target of miR-223. Furthermore, serum miR-223 levels were decreased in female patients with PAH associated with congenital heart disease. Our study provides the first evidence that miR-223 can regulate PASMC proliferation, migration, and actomyosin reorganization through its novel targets, RhoB and MLC2, resulting in vascular remodeling and the development of PAH. It also highlights miR-223 as a potential circulating biomarker and a small molecule drug for diagnosis and treatment of PAH.

Chronic hypoxia-induced pulmonary arterial hypertension (PAH) is characterized by persistent elevation of pulmonary arterial pressure due to pulmonary vasoconstriction and pulmonary vascular remodeling accompanied by right ventricular hypertrophy^1^. It is well accepted that the primary cellular mechanism underlying vascular remodeling is the excessive proliferation and migration of pulmonary arterial smooth muscle cells (PASMC)^2^,^3^. However, the exact molecular mechanisms that regulate vascular proliferation are incompletely understood.

Currently, signaling pathways, such as the Rho-kinase pathway, are implicated as important factors in the pathogenesis of PAH^1^. RhoA has been widely reported as a regulator of actomyosin-related processes such as cell adhesion, migration and proliferation^4^. Recent data show that RhoB is highly expressed in lung tissue and is involved in cellular responses to oxidative stress^5^,^6^. Decrease in RhoB markedly inhibits hypoxia-induced endothelial permeability and growth responses in pulmonary vascular cells. Moreover, genetic deletion of RhoB attenuates development of chronic hypoxia-induced vascular remodeling and pulmonary hypertension in mice^5^.

MicroRNAs (miRNAs) have been identified as essential modulators of a variety of genes and cellular processes^7^. Several miRNAs have been reported to regulate different steps in the process of angiogenesis and vascular remodeling^8^. miR-21 negatively regulates programmed cell death 4 (PDCD4) promoting PASMC proliferation and migration^9^,^10^. Downregulation of miR-204 correlates with PAH severity and accounts for the pro-proliferative and anti-apoptotic phenotypes of PASMC in PAH^11^. miR-143 and miR-145 have been demonstrated to play critical roles in vascular smooth muscle cell phenotype switching^12^,^13^. Decreased expression of miR-124 affects the proliferation, migration and inflammatory phenotype of both PASMC and pulmonary vascular fibroblasts^14^,^15^. Alterations of the miR-210–iron–sulfur cluster assembly homolog 1/2 (ISCU1/2) regulatory axis can cause Fe-S deficiencies *in vivo* and is crucial to the development of PAH^16^. miR-17~92 inhibits PDZ and LIM domain 5 (PDLIM5) to induce the transforming growth factor-beta 3/ SMAD family member 3 (TGF-beta3/SMAD3) pathway, contributing to the pathogenesis of PAH^17^. Treatment with antagomiR-20a restores the functional levels of bone morphogenetic protein receptor II (BMPR2) in pulmonary arteries and prevents the development of vascular remodeling^18^. miRNAs are now recognized as participants in multiple disease pathways in the pulmonary vasculature, however their true importance in PAH is just beginning to emerge^19^.

miR-223 was initially described as a myeloid-specific miRNA^20^,^21^ and as a biological marker for several tumoral processes^22^,^23^,^24^. It has also been reported to play an important role in neuroprotection^25^, and as a marker of damage to both skeletal^26^ and cardiac muscle tissues^20^. Recent studies have indicated that miR-223 is an anti-angiogenic miRNA that prevents endothelial cell proliferation, migration and sprouting by targeting β1 integrin^27^. In this study, we elucidated the role of miR-223 in pulmonary vascular responses to hypoxia *in vitro* and *in vivo*, and identified a RhoB/ myosin light chain-2 (MCL2)-dependent role of miR-223 in PASMC and its clinical importance as a potential biomarker for the diagnosis of pulmonary arterial hypertension associated with congenital heart disease (CHD-PAH).

## Results

### Hypoxia decreases miR-223 expression in lungs, pulmonary artery and pulmonary arterial smooth muscle cells

**Figure 1:**
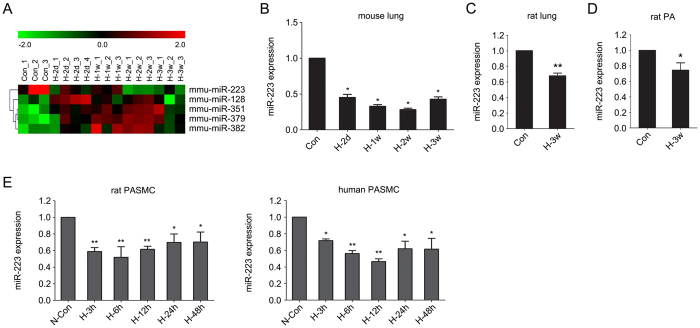
miR-223 is downregulated by hypoxia in PASMC. (**A**) Differential expression of candidate miRNAs in mouse lung tissue exposed to normoxia (21% O_2_)(Con) or hypoxia (10% O_2_) for 2 days (H-2d), 1 week (H-1w), 2 weeks (H-2w) and 3 weeks (H-3w) (n = 3 or 4 for each group). Cluster analysis of miRNA expression in a 1040 miRNA microarray set measured from individual specimens (*p* < 0.05 compared with normoxic controls). Validation of miR-223 downregulation by hypoxia in mouse lungs (**B**), rat lungs, n = 8 (**C**) and rat pulmonary arteries (PA), n = 8 (**D**) was performed by qRT-PCR. (**E**) Rat and human PASMC were exposed to normoxia (21% O_2_) (N-Con) or hypoxia (H, 3% O_2_) (n = 3) for up to 48 h. The relative levels of miR-223 were measured by real-time PCR using SNORD44 (for human sample) or snoRNA202 (for mouse or rat sample) as an internal control. Experiments were performed in triplicate. Bar charts show the relative expression level by normalizing to the normoxia control (N-Con). Data are shown as means ± SE, *p < 0.05, **p < 0.01.

To identify miRNAs that are altered during the development of chronic hypoxia-induced pulmonary hypertension, we compared miRNA expression levels in normoxic and chronically hypoxic mouse lungs by microarray analysis. The results revealed that standing out from 1040 miRNAs measured, there were five significantly changed miRNAs between normoxia and hypoxia (*p* < 0.01) and miR-223 (also called miR-223-3p) was the most significantly downregulated miRNA (Fig. 1A). An independent quantitative real-time PCR (qRT-PCR) assay confirmed the decreased expression of miR-223 in mouse lungs in response to hypoxia (Fig. 1B). The decrease in miR-223 was also observed in rat lungs and in pulmonary arteries (PA) following 3-weeks of hypoxia (Fig. 1C,D, respectively).

To determine whether miR-223 expression in PASMC parallels expression in lungs of hypoxic mice, we isolated PASMC from Sprague-Dawley rats and exposed them to hypoxia. We observed a 50% decrease in miR-223 levels, with a maximal decrease after 6 to 12 hours of hypoxia (Fig. 1E, left panel). Comparable results were obtained when expression of miR-223 was studied in human PASMC (hPASMC) under hypoxic conditions (Fig. 1E, right panel). These results point to miR-223 as a miRNA that is decreased by hypoxia in PASMC.

### MiR-223 represses pulmonary smooth muscle cell proliferation and migration

**Figure 2:**
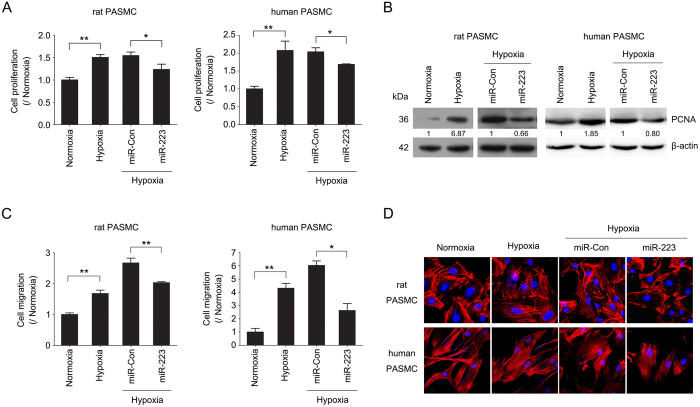
miR-223 inhibits PASMC proliferation, migration and stress fiber formation. Rat and human PASMC were transfected with mimic control (miR-Con) or miR-223 mimic (miR-223) and exposed to hypoxia (3% O_2_) or normoxia for 24 h in triplicate. Cell proliferation was determined by EdU incorporation assay (**A**) and PCNA immunoblotting (**B**). Data are shown as means ± SE (n = 3). (**C**) Transwell migration assay showing the migration of rPASMC or hPASMC with miR-223 overexpression. Bar charts representing relative migrated cells after 24 h. Data (mean ± SE) is derived from an average of three independent experiments, with five visual fields in triplicate samples. (**D**) F-actin in cells was stained with TRITC-phalloidin and visualized by confocal microscopy. *p < 0.05, **p < 0.01.

Pulmonary vascular remodeling in response to chronic hypoxia is principally associated with the excessive proliferation and migration of PASMC^2^,^28^. To assess the role of miR-223 in modulating cell proliferation, cells were transfected with miR-223 mimic, which increased miR-223 levels in cultured rPASMC and hPASMC (Supplementary Figure S1). EdU incorporation assay and PCNA immunoblotting showed that miR-223 overexpression significantly prevented the increase in cell proliferation induced by hypoxia (Fig. 2A,B). To confirm the consequences of miR-223 on cell proliferation, EdU incorporation assay and PCNA immunoblotting were also performed in cells transfected with miR-223 inhibitor. Conversely, miR-223 inhibition augmented the proliferation of PASMC under normoxic conditions (Supplementary Figure S2A, B).

Furthermore, enhanced expression of miR-223 also inhibited hypoxia-induced increase in cell migration in both rPASMC and hPASMC, as measured using a chamber invasion assay of cells passing through a transwell filter (Fig. 2C). Moreover, the altered migration in miR-223-expressing cells was associated with a decrease in stress fiber levels, a feature reflected by the higher F-actin content in cells that have active actomyosin contraction (Fig. 2D). All the results above indicate that miR-223 is involved in the proliferative and migratory responses in PASMC to hypoxia.

### MiR-223 attenuates chronic hypoxia-induced pulmonary vascular remodeling

**Figure 3:**
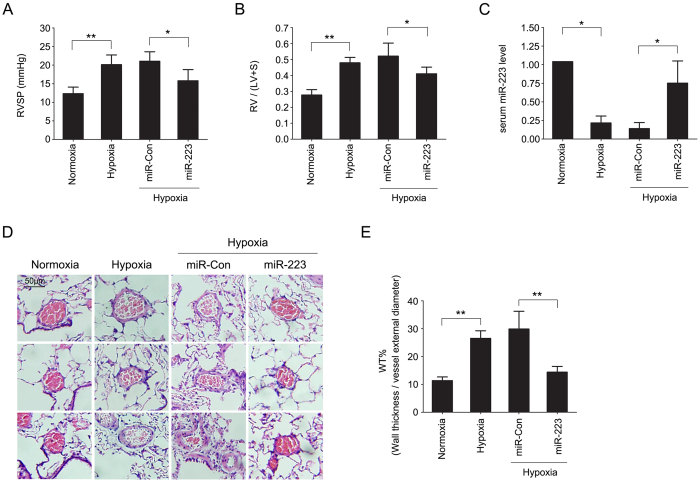
miR-223 attenuates chronic hypoxia-induced pulmonary vascular remodeling. Chronic hypoxia-induced PAH in a rat model was established by 3 weeks of hypoxic treatment. Rats were injected with either agomir control (miR-Con) or agomir specifically expressing miR-223 (miR-223). Right ventricular systolic pressure (RVSP) (**A**) and right ventricular hypertrophy [RV/ (LV + S)] (**B**) are shown in bar charts. Data are shown as means ± SE (n = 8). (**C**) The relative levels of serum miR-223 in each rat group were measured by real-time PCR using rnomiR-16-5p as a normalization control. Data are shown as means ± SE (n = 8). (**D,E**) The wall thickness of small and medium sized pulmonary arteries was assessed from HE stained lung sections. Scale bar = 50 μm. Bar charts showing the percentage of wall thickness to vessel external diameter (WT%) of each rat group. Data are shown as means ± SE (n = 8), *p < 0.05, **p < 0.01.

To confirm our findings *in vivo*, we determined the effect of administering an agomir of miR-223 or an unrelated agomir control on right ventricular systolic pressure (RVSP) and pulmonary vascular muscularization in rats exposed to chronic hypoxia. Following 3 weeks of hypoxia exposure, the RVSP in wild-type rats increased from 12.33 ± 1.75 mmHg to 20.14 ± 2.58 mmHg (*p* < 0.01). Rats with miR-223 agomir treatment showed a significantly attenuated increase in RVSP in hypoxia (15.81 ± 3.00 mmHg in miR-223 compared with 21.06 ± 2.54 in miR-Con, *p* < 0.05) (Fig. 3A). Coincident with the effects on pulmonary vascular pressure, the ratio of right to left ventricle plus septum weight [RV/(LV + S)] in rats treated with miR-223 agomir also showed an attenuated increase in hypoxia (Fig. 3B). RVSP or RV/(LV + S) in rats treated with agomir control were not different compared with hypoxic control group. Next, we measured miR-223 levels in peripheral blood from each experimental group by qPCR. Serum miR-223 levels were significantly decreased in hypoxia-exposed rats (*p* < 0.05, compared with normoxia group) and the levels were increased with miR-223 agomir administration (*p* < 0.05, compared with miR-Con group in hypoxia) (Fig. 3C). As pulmonary vascular remodeling is a characteristic feature of PAH^19^,^29^, we noted that the delivery of synthetic miR-223 agomir markedly reduced hypoxia-increased medial wall thickness of small and medium sized pulmonary arteries (Fig. 3D,E). Our results show that pulmonary artery pressure and distal arteriolar muscularization following chronic hypoxia were significantly decreased when level of miR-223 was preserved.

### MiR-223 targets RhoB and MLC2 in pulmonary arterial smooth muscle cells

**Figure 4:**
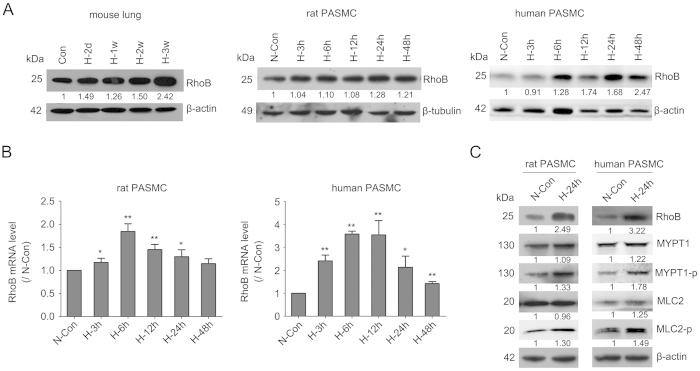
Hypoxia upregulates RhoB/Rho-kinase signaling in PASMC. (**A**) Representative western blot for hypoxia-induced increase of RhoB in mouse lungs, rat PASMC and human PASMC (n = 3). β-actin was used as a loading control. (**B**) RhoB mRNA levels in hypoxia-exposed rat and human PASMC were evaluated by qRT-PCR using β-actin as an internal control. Bar charts showing the relative expression level by normalizing to the normoxia control (N-Con). Data are shown as means ± SE (n = 3). (**C**) Expression of RhoB/Rho-kinase signaling pathway related components in rat and human PASMC in response to hypoxia was determined by western blotting (n = 3). *p < 0.05, **p < 0.01.

To determine how miR-223 regulates PASMC proliferation, migration and consequently pulmonary vascular remodeling, we identified the miR-223 target proteins involved in these processes. A negative correlation between the expression of miR-223 and RhoB protein was detected in mouse lungs, as well as in rat and human PASMC exposed to hypoxia (Fig. 4A). The mRNA level of RhoB in both hypoxia-treated rPASMC and hPASMC was also negatively correlated with miR-223 expression (Fig. 4B).

Rho-kinase (ROCK) is a direct downstream effector of Rho GTPase (such as RhoA and RhoB). It phosphorylates the regulatory subunit of myosin phosphatase, known as MYPT1, and inhibits its phosphatase activity. This inhibition increases phosphorylation of MLC, which is thought to induce Rho-mediated assembly of stress fibers and focal adhesions^30^. Hypoxia is a common stimulus for activating Rho/ROCK and thereby facilitates the phosphorylation of MYPT1 and MLC^30^,^31^. Here, we observed increased levels of phosphorylated MYPT1 and MLC2 in both rPASMC and hPASMC following exposure to hypoxia (Fig. 4C).

**Figure 5:**
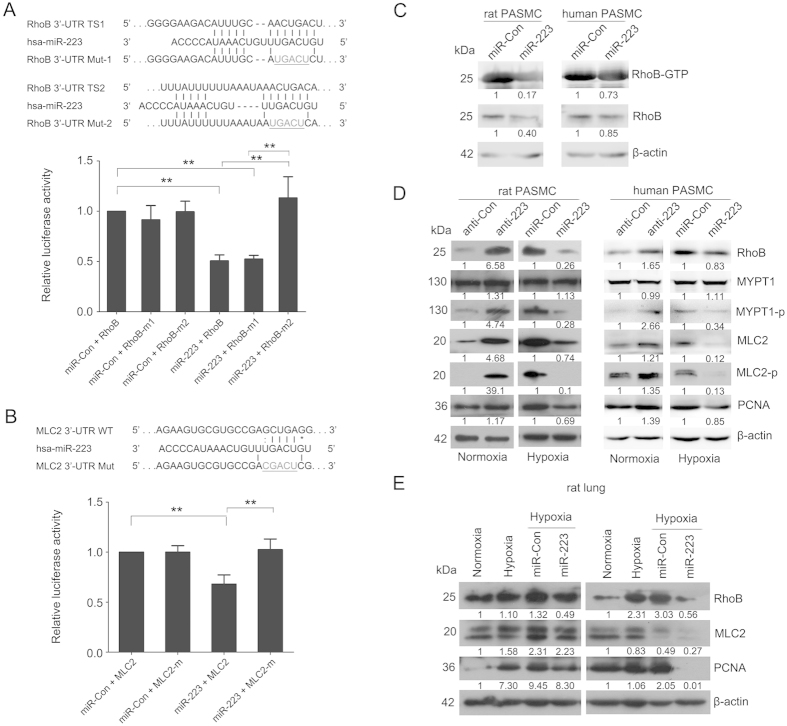
Identification of RhoB and MLC2 as direct targets of miR-223. Luciferase reporter gene assay on the interaction between miR-223 and 3′-UTR of RhoB (**A**) and MLC2 (**B**) in HEK293 cells. Upper panel: putative miR-223 binding sites in the 3′-UTR of RhoB or MLC2 along with the mutation sites. TS1,target site 1 (a pooly conserved binding site); TS2, target site 2 (a conserved binding site); Bottom panel: 3′-UTR luciferase reporter assay with targets and their mutant along with miR-223/miR-Con overexpressing vectors. Bar charts of luciferase reporter analysis represent means ± SE (n = 3), firefly luciferase activity was normalized to renilla luciferase activity. (**C**) Active RhoB proteins in miR-223 or miRNA control overexpressing PASMC were pulled down by rhotekin-RBD beads and detected by western blotting analysis using anti-RhoB antibody (n = 3). (**D**) Expression of RhoB/Rho-kinase signaling pathway related components in PASMC, with overexpression or inhibition of miR-223, was measured by western blotting (n = 3). (**E**) Expression of RhoB, MLC2 and PCNA in lungs of hypoxia-exposed rats administered agomirs miR-223/miR-Con was determined by western blotting (n = 3). β-actin was used as a loading control for the above western blot analysis. Two representative blots are shown. *p < 0.05, **p < 0.01.

Using an online tool (TargetScan, www.targetscan.org), we found two potential miR-223 binding sites within RhoB 3′-UTR (Fig. 5A). In order to validate RhoB as a direct target of miR-223, we performed a reporter gene assay to ascertain the interactions between miR-223 and RhoB 3′-UTR. Co-transfection of HEK-293 cells with pre-miR-223 and a luciferase construct containing the two putative miR-223 binding sequences within the 3′-UTR of RhoB resulted in a significant decrease (about 50%, *p* < 0.01) in luciferase activity, indicating direct interaction between them. Notably, the two target sites when tested in the assay exhibited substantial differences in their susceptibility to miR-223 mediated repression. Mutation of the downstream binding site (Mut 2) completely antagonized the inhibitory effect of miR-223 in the luciferase assay. However, effect was unchanged when the target site 1 sequence was mutated (Fig. 5A). The results above clearly show that RhoB is a direct target of miR-223 in PASMC. Rho GTPase activation assay showed that miR-223 not only repressed the expression of RhoB but also markedly reduced its activity (Fig. 5C). miR-223 inhibition also enhanced the phosphorylation of MYPT1 and MLC2, partially mimicking the effects of hypoxia. Conversely, overexpression of miR-223 reduced the levels of phosphorylated MYPT1 and MLC2 (Fig. 5D). Notably, a negative correlation of total MLC2 expression and miR-223 levels was found in rPASMC and hPASMC (Fig. 5D). miRNA target prediction with FINDTAR3 indicated a potential miR-223 binding site in the 3′-UTR of MLC2. Reporter gene assay revealed a direct interaction between miR-223 and the 3′-UTR of MLC2, which was confirmed by binding site mutants in reporter assay (Fig. 5B). Results above demonstrate that miR-223 contributes to hypoxia-induced phosphorylation of MYPT1 by targeting RhoB and more importantly, has a direct inhibitory effect on MLC2 expression. In addition, we measured the protein levels of these two targets in rats with hypoxia-induced PAH with miR-Con and miR-223 agomir treatment. As shown in Fig. 5E, both RhoB and MLC2 proteins in hypoxia-treated rat lungs were increased, but to a significantly lesser degree in rats treated with synthetic miR-223 agomir when compared to agomir control. We observed similar results with the cell proliferative marker PCNA, indicating that miR-223 has an anti-proliferative effect in experimental PAH.

### Serum miR-223 is a potential circulating biomarker for PAH

**Figure 6:**
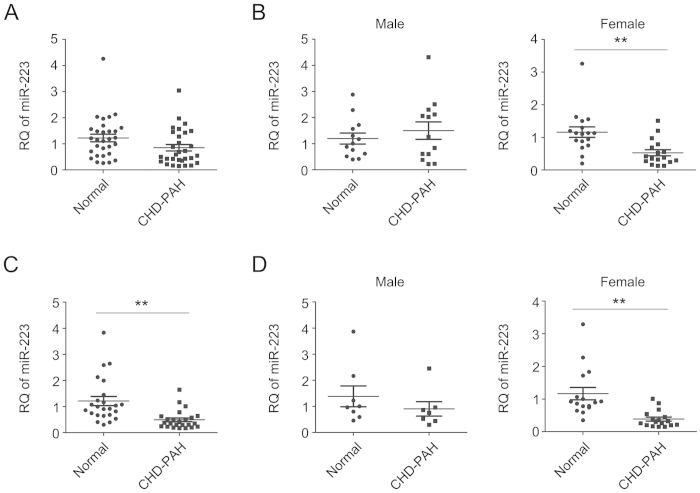
Serum miR-223 as a potential circulating biomarker for CHD-PAH. Expression of serum miR-223 was measured by qRT-PCR in healthy human donors (normal) and CHD-PAH patients. Levels of miR-223 in 30 normal versus 30 CHD-PAH patients (**A**), 13 male normal versus 13 male CHD-PAH patients, and 17 female normal versus 17 female CHD-PAH patients (**B**), a second group of 24 normal versus 24 patients (**C**), 7 male normal versus 7 male patients, and 17 female donors versus 17 female patients (**D**), respectively were normalized to hsa-miR-16-5p and represented in scatter plots. Data are shown as means ± SE, **p < 0.01.

As serum miRNAs are currently used as potential biomarkers for human diseases^32^,^33^,^34^, we wondered if circulating miR-223 levels were decreased in patients with PAH akin to that in experimental PAH and human PASMC. First, we measured miR-223 levels in sera from 30 patients with CHD-PAH, mainly with ventricular septal defect (VSD) or atrial septal defects (ASD) (Supplementary Table 1). We found that serum miR-223 levels in patients were lower than that in healthy donors but with no significant difference (*p* = 0.058) (Fig. 6A). As miR-223 locus is located on the X chromosome, a new study was carried out on 13 male CHD-PAH patients/ healthy donors or 17 female CHD-PAH patients/ healthy donors to determine whether gender differences exist (Supplementary Table S1). Our results show that the levels of circulating miR-223 in CHD-PAH manifest a gender related difference. Serum levels of miR-223 in female CHD-PAH patients were significant lower than that in healthy female donors, but there was no difference between male CHD-PAH subjects and control subjects (Fig. 6B). Another study on 7 male and 17 female CHD-PAH patients/ healthy donors (Supplementary Table S2) showed significant decrease in circulating miR-223 levels in CHD-PAH patients and confirmed the gender-related alteration (Fig. 6C,D). These results are of great clinical significance as it highlights miR-223 as a potential diagnostic circulating biomarker in female CHD-PAH patients.

## Discussion

**Figure 7:**
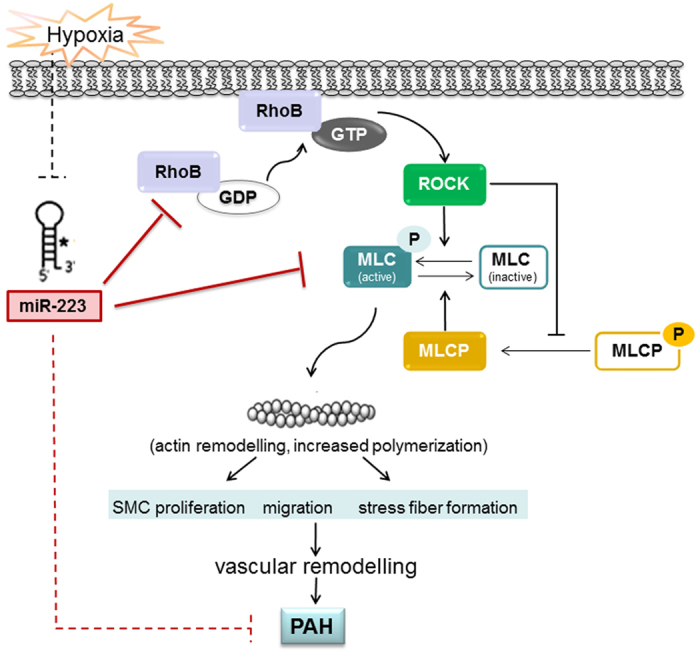
Schematic model showing the role of miR-223 in pulmonary arterial hypertension in response to hypoxia. miR-223 attenuates hypoxia-induced smooth muscle cell proliferation, migration and actomyosin reorganization, and the consequent vascular remodeling and pulmonary hypertension by regulating the RhoB/MLC pathway.

This study reveals that miR-223 is rapidly downregulated in response to hypoxia in PASMC and is critical to chronic hypoxia-associated pulmonary vascular pathology, such as smooth muscle cell proliferation, migration and actomyosin reorganization, which ultimately results in vascular remodeling and distal arteriole muscularization. We elucidate a RhoB/ROCK-associated regulatory pathway that is suppressed by miR-223 and the decrease of miR-223 in hypoxia leads to pathologic changes in PASMC (as summarized in Fig. 7). This study identifies miR-223 as a potential target in experimental PAH and the possible benefits of miR-223 agomir therapy in its treatment. It also suggests a potential use of miR-223 levels as a circulating biomarker for clinical diagnosis in women with CDH-PAH.

Hypoxic pulmonary vasoconstriction helps to maintain oxygen supply within physiological limits by diverting blood to the poorly ventilated areas of the lung^35^. A specific and still growing list of miRNAs, so-called “hypoxamirs”, are characterized to be dynamically regulated by hypoxia^36^. In this study, we discovered a decrease in expression of miR-223 in hypoxia-induced PAH mouse lungs, pulmonary artery and isolated PASMC. In an early study, Caruso *et al.* reported that miR-223 was decreased in chronic hypoxia-treated PAH rat lungs^37^. Recently, Shi *et al.* reported that miR-223 antagonizes angiogenesis by abrogating VEGF and bFGF-induced cell proliferation, migration and tube formation in vascular endothelial cells^27^. Rangrez *et al.* found that overexpressing miR-223 in aortic vascular smooth muscle cells (VSMCs) increased proliferation and markedly enhanced cell migration^38^, demonstrating miR-223 may function in a tissue-specific manner. Our data show that increasing the expression of miR-223 abrogated the hypoxia-induced increase in proliferation, migration, as well as stress fiber assembly in both rPASMC and hPASMC. Overexpression of miR-223 using agomir antagonized the hypoxic effects on pulmonary artery pressure and distal arteriolar muscularization *in vivo*. DNA-damage induced PARP-1 has been shown to be involved in PAH-PASMCs and interestingly baseline DNA-damage was demonstrated to be higher in PAH patients and their relatives^39^. During the preparation of this manuscript, Meloche *et al.* most recently showed that miR-223 was downregulated in PAH-PASMCs and lungs of monocrotaline-treated PAH rats. They reported that miR-223 played anti-proliferative and pro-apoptosis roles in PAH-PASMC by directly repressing PARP-1^40^. Our study demonstrates that miR-223 regulation directly targets RhoB and MLC2 to affect vascular remodeling and hypoxia-induced pulmonary hypertension.

Both RhoA and RhoB appear to cooperate in hypoxia-induced cytoskeletal remodeling, but RhoB plays a unique role in the regulation of vascular proliferation responses to hypoxia^5^. Recent reports implicate RhoB as a regulator of hypoxia-induced cell proliferation in both human pulmonary artery endothelial cells and hPASMC. Moreover, RhoB deficiency in mice markedly attenuated development of chronic hypoxic pulmonary hypertension, despite compensatory expression of RhoA in the lung^5^. Stothard *et al.* have reported that RhoB protein levels and activity in hPASMC rapidly increased to maximum between 2 to 4 hours in 2% O_2_, while RhoA mRNA and protein levels remained relatively unchanged with a small increase in its activity. However in this study, we found that RhoB mRNA levels in hPASMC, when exposed to a milder hypoxic condition (3% O_2_), reached a maximum between 6 to 12 hours, with protein levels continuing to rise until 24 hours. In cultured rPASMC, RhoB gene and protein expression increased steadily after hypoxic exposure and reached maximum levels at 6 h and 24 h respectively. These hypoxia-induced changes in RhoB were opposite to the effects on miR-223, pointing to a negative relationship between them.

The correlation between the expression of RhoB and miR-223 was ascertained in primary culture of PASMC and in an *in vivo* PAH rat model by both miR-223 overexpression and/or knockdown approaches. It was previously reported that the 3′-UTR of RhoB could be targeted by hsa-miR-223 in human cell lines^41^. Here we further corroborated RhoB as a direct target of rno-miR-223 in rat PASMC. TargetScan predicted RhoB having two miR-223 target sites and defined site 1 as poorly conserved and site 2 as a conserved site. Reporter gene analysis revealed that mutation of site 2 resulted in complete loss of the inhibitory activity of miR-223, while site 1 mutation showed no activity change compared to wild type construct, although it indicates that there may be more bases pairing with miR-223. The results fit well with a recent report that hsa-miR-223 interacts with RhoB, in which the authors proposed that some AU-rich motif located upstream of distal miR-223-binding site enhances the miRNA function, independent of the miRNA target sequence being tested^41^.

Previous studies have demonstrated that constitutively activated Rho proteins protect against the disruption of stress fibers and control actomyosin contractility in both smooth muscle and non-muscle cells^42^. MLC phosphorylation is a critical step that leads to activation of myosin ATPase and subsequent smooth muscle cell contraction in response to hypoxia. MLC is phosphorylated by MLC kinase (MLCK) and de-phosphorylated by MLC phosphatase (MLCP). ROCK, a specific downstream effector of Rho GTPase, increases the levels of phosphorylated MLC by direct phosphorylation and also through the inhibition of MLCP by phosphorylating its regulatory subunit (MYPT1)^30^,^43^. Our data confirm that hypoxia resulted in a significant increase in phosphorylation of MYPT1 and MLC. Total MYPT1 and MLC2 protein levels were not different when compared with that in normoxic conditions. Overexpression of miR-223 caused a remarkable decrease in MLC2 protein levels, while miR-223 inhibition increased the expression of MLC2. Further, we demonstrated the 3′-UTR of MLC2 mRNA was post-transcriptionally regulated by miR-223. Our study has uncovered for the first time a regulatory miRNA that directly targets RhoB and MLC2 in the Rho/ROCK signaling pathway (Fig. 7).

Due to emerging evidence for a role of Rho proteins in pulmonary hypertension, specific and potent modulators of various steps of Rho GTPase signaling pathway are becoming promising means for pharmacological intervention. ROCK inhibitors, such as Fasudil and Y-27632, are examples of two widely used chemical compounds that have been developed to treat hypoxia-induced pulmonary vascular remodeling. The success of the ROCK inhibitors in the treatment of vascular disease has encouraged the use of Rho pathway inhibition in clinical studies. On the other hand, there are few effective inhibitors directly targeting Rho GTPases, likely owing to the lack of optimal structural information on interaction with individual GTPase to achieve specificity. However, the dynamic changes of Rho GTPase in disease states underline the need to develop effective inhibitors for therapeutic applications. Here, we discovered that treatment with miR-223 agomir markedly attenuates chronic hypoxia-induced pulmonary vascular remodeling *in vivo*, due to its post-transcriptionally inhibitory effects on RhoB and MLC2 expression. Therefore, a small molecular drug, like miR-223, may improve efficacy by targeting the RhoB/Rho Kinase/MLC2 signaling in pulmonary vascular diseases.

There is increasing evidence that circulating miRNAs may be informative in disease diagnosis and have a potential to be used as biomarkers of disease^44^,^45^. Stable miRNAs in the circulation are speculated to exist in microvesicles or exosomes and apoptotic bodies, which are secreted into the circulation and are extremely stable in the plasma or serum^46^,^47^. miR-223 is currently of interest as its levels in circulation are inversely correlated with the risk of developing coronary heart disease, diabetes and myocardial infarction^48^,^49^,^50^. A recent study on circulating miRNAs as potential markers for pulmonary hypertension by using a microarray approach identified that plasma miR-223 levels were decreased in these patients^34^. In our study, we uncovered that there are gender differences in downregulation of serum levels of circulatory miR-223 in patients with CHD-PAH. However, no gender difference was observed in healthy donors. Torres *et al.* reported that patients with chronic obstructive pulmonary disease (COPD) also manifested gender related differences in some plasma inflammatory cytokines^51^. Circulating miRNAs are speculated to be released from the damaged lungs or vessels in patients with pulmonary hypertension^34^. Recently, Ma reported that miR-27b was greatly up-regulated in the lung of CHD-PAH patients and correlated well with preoperative mean pulmonary arterial pressure^52^. Our results point to a gender-specific response in circulating miR-223 in pulmonary hypertension associated with CHD. It still remains unknown if there is a relationship between miR-223 reduction in female serum and gene regulatory abnormality on X chromosome in CHD-PAH patients.

The data presented are based on a limited number of subjects, and the results need to be further validated in a larger cohort of the disease. We have not explored the miR-223 expression and RhoB/ROCK/MLC pathway in pulmonary artery endothelial cells (PAECs), the abnormality of which also contributes to the pathology of pulmonary arterial hypertention. It will be of great interest to study whether miR-223 agomir shows better effect by intratracheal nebulization.

## Materials and Methods

### Cell culture

Human HEK293 cells were purchased from American Type Culture Collection (ATCC, Manassas, VA) and maintained in Dulbecco’s modified Eagle’s medium (DMEM) supplemented with 10% fetal bovine serum (FBS). Human pulmonary arterial smooth muscle cells (hPASMC) were purchased from Lonza (Walkersville, MD) and cultured in SmGM-2 smooth muscle growth media consisting with smooth muscle basal medium, 5% FBS, 0.5 ng/ml human recombinant epidermal growth factor, 2 ng/ml human recombinant fibroblast growth factor, 5 μg/ml insulin, and 50 μg/ml gentamicin.

Rat pulmonary arterial smooth muscle cells (rPASMC) were dissociated from the pulmonary artery of Sprague-Dawley rats (male, 6 weeks). Briefly, a segment of pulmonary artery just proximal to the lung entry was removed under aseptic conditions and cleaned from connective and fat tissues; The media of the pulmonary artery, dissected from the adventitia and intima, was subjected to anenzymatic digestion in buffer containing 1 mg/ml collagenase (Worthington Biochemical Corporation) and 0.5 mg/ml Elastase type IV (Sigma-Aldrich) for 60 min at 37 °C. For hypoxic treatment, PASMC were placed in a special hypoxia incubator infused with a gas mixture of 5% CO_2_ and nitrogen to obtain 3% oxygen concentration. Oxygen concentration was monitored continuously (Forma 3130; Thermo Scientific).

### miRNA microarray assay

Microarray assay of mature miRNAs was performed by the LC Sciences Microarray Service (LC Sciences, Houston, TX). Briefly, total RNA (5 μg) was size fractionated (<300 nucleotides) by using a YM-100 Microcon centrifugal filter (Millipore) and the small RNAs (<300 nt) isolated were polyadenylated with poly(A) polymerase. An oligonucleotide tag was then ligated to the poly(A) tail for later fluorescent dye staining. Each sample was hybridized to a microarray that contained 1040 mature mouse miRNA probes from the Sanger miRBase 16.0 (http://www.sanger.ac.uk/Software/Rfam/mirna/). After RNA hybridization, tag-conjugating Cy3 and Cy5 dyes were circulated through the microfluidic chip for dye staining. Fluorescence images were collected using a laser scanner (GenePix 4000B, Molecular Device) and digitized using Array-Pro image analysis software (Media Cybernetics).

Data were analyzed by first subtracting the background and then normalizing the signals using a LOWESS filter (Locally-Weighted Regression) for two color experiments. Transcripts were determined as detectable if their signal intensity was higher than 3 times the background standard deviation, spot CV (standard deviation/signal intensity) was <0.5, and transcripts had at least 50% of repeating probe signals above the detection level. *P*-values of the ANOVA were calculated. The miRNAs significantly expressed in hypoxia-treated mice compared with normoxic control (*p* values <0.05) were selected for cluster analysis according to a hierarchical method, which was performed with average linkage and Euclidean distance metrics. The clustering plot was generated using TIGR MeV (Multiple Experimental Viewer) software from the Institute for Genomic Research.

### miR-223 overexpression and inhibition

Chemically synthesized miRNA mimics or inhibitors to overexpress or inhibit miR-223 and each unrelated negative control (miR-Con or anti-Con), were purchased from Ribobio (Guangzhou, China). Cells at 70% confluence after overnight culture on petri dishes were transfected with miR-223 mimic, mimic control (miR-Con) (50 nmol/L), or miR-223 inhibitors (anti-223), inhibitor control (anti-Con) (100 nmol/L) by Lipofectamine 2000 (Invitrogen). After 6 h transfection, medium was changed, and the cells were cultured for 24 h and then exposed to either hypoxic or normoxic conditions for 24 h or 48 h.

### Quantitative RT-PCR

Total RNA of tissue and serum sample was extracted with RNAiso Plus (TaKaRa Biotechnology, China). For mRNA evaluation, 1 μg of total RNA was reverse-transcribed using M-MLV Reverse Transcriptase (TaKaRa) with oligo(dT)_18_ plus random hexamer primers (Promega). qRT-PCR was performed with gene specific primers and SYBR Green PCR Master Mix (Applied Biosystems, Foster City, CA) on ABI StepOneplus real-time PCR System (Applied Biosystems). The expression level of each gene was normalized to internal control β-actin gene and the expression level of each mRNA was calculated using the 2^(−ΔΔCt)^ method. For miRNA assay, the mature miR-223 expression level was determined according to S-Poly(T) method, as we previously reported^53^,^54^. The miR-223 expression level was normalized to SNORD44 (in human tissue sample), rno-miR-16-5p (in rat serum sample) or snoRNA202 (in mice or rat tissue sample) and calculated using the 2^(−ΔΔCt)^ method. The miR-223 expression level in human serum samples was normalized to hsa-miR-16-5p and calculated using the 2^(−ΔCt)^ method. Each PCR reaction was analyzed in triplex tubes. Primers used for reverse transcriptionand quantitative PCR were listed as follows: human RhoB: 5′-CGG ACT CGC TGG AGA ACA-3′ (forward) and 5′-GAG GTA GTC GTA GGC TTG GAT-3′ (reverse); rat RhoB: 5′-CGG ACT CTC TCG AGA ACA-3′ (forward) and 5′-GAG GTA GTC ATA GGC TTG GAT-3′ (reverse); rat β-actin: 5′-AAA GAC CTG TAC GCC AAC AC-3′ (forward) and 5′-GTC ATA CTC CTG CTT GCT GAT-3′ (reverse); human β-actin: 5′-AGA GAT GGC CAC GGC TGC TT-3′ (forward) and 5′-ATT TGC GGT GGA CGA TGG AG-3′ (reverse); miR-223: 5′-GTG CAG GGT CCG AGG TCA GAG CCA CCT GGG CAA TTT TTT TTT TTG G-3′ (RT) and 5′-GGT GTC AGT TTG TCA AAT ACC C-3′ (forward); miR-16: 5′-GTG CAG GGT CCG AGG TCA GAG CCA CCT GGG CAA TTT TTT TTT TTC GCC AA-3′ (RT) and 5′- CCG GGT AGC AGC ACG TAA ATA-3′ (forward); SNORD44: 5′-GTG CAG GGT CCG AGG TCA GAG CCA CCT GGG CAA TTT TTT TTT TTA GTC AG-3′ (RT) and 5′-TGG CCT GGA TGA TGA TAA GCA-3′ (forward); snoRNA202: 5′-GTG CAG GGT CCG AGG TCA GAG CCA CCT GGG CAA TTT TTT TTT TTC ATC AG-3′ (RT) and 5′- GTA CTT TTG AAC CCT TTT CCA T-3′ (forward).

### Cell proliferation assay

Cell proliferation was determined by EdU incorporation assay and PCNA immunoblotting. EdU labeling was performed by using the EdU Assay Kit (Ribobio, Guangzhou, China) following the procedures suggested by the manufacturer. Briefly, approximately 1 × 10^4^ cells were seeded in triplicates in 48-well plates, and the cells were cultured for 24 h and then exposed to either hypoxic or normoxic conditions for 24 h, then exposed to 20 μmol/L EdU for 24 h at 37 °C. The cells were then fixed in 4% paraformaldehyde for 30 min at room temperature and permeabilized in 0.5% Triton X-100 for 10 min. Cells were washed with PBS, and each well was incubated with 200 μl 1× Apollo reaction cocktail for 30 min. DNA was then stained with 1 μg/ml DAPI (200 μl per well) for 5 min and imaged under a fluorescent microscope. All data are shown as a percentage of normoxia controls. PCNA Western blot studies were performed as described in Western blotting. All experiments were performed in triplicate.

### Transwell Cell migration assay

Cells were seeded on the upper chambers of a 24 well format cell culture insert with 8 μm pores (FALCON, Le Pont De Claix, France). Upper Medium was replaced by starvation medium (containing 0.5% FBS), while the lower chamber contained medium with 5% FBS. 24 h later, the number of transmigrated cells was counted after trypsin treatment. Data were expressed as percentage of control. Cell migration data were obtained from three independent experiments.

### Localization of F-Actin

F-actin was visualized by affinity-fluorescence methods. Fluorescent emissions from TRITC-phalloidin (Cytoskeleton, USA) bound to F-actin were analyzed by confocal microscopy at the same level of laser intensity for all the studied samples.

### *In vivo* chronic hypoxic animal model

Animal experiments were carried out in accordance with the guidelines approved by the Ethics Committee of Shenzhen University. The permit number of experiment animal for this study is SCXK (Yue) 2008-0002. Chronic hypoxia-induced PAH mouse model was performed as previously described^55^. Lungs from PAH mice were stored at −80 °C prior to use. Rats for *in vivo* studies were randomized into four groups (n = 8 each): 1) a normoxic control group, 2) a hypoxic control group, 3) a hypoxic group injected with agomir control, and 4) a hypoxic group injected with miR-223 agomir (Ribobio). miR-223 agomirs (30 nmol), agomir control (30 nmol), or the normal saline (NS) were injected intravenously (tail vein, 0.3 ml) at day 7 and 14. Degree of PAH were assessed by measuring right ventricular systolic pressure (RVSP) and the ratio of right to left ventricle plus septum weight [RV/(LV + S)] at day 21 as described^56^. After RVSP recording, whole blood were collected via right ventricular puncture, filled in 1.5 ml tubes, centrifuged for 3,000 × g for 10 min at 4 °C, and serum collected for detection of miR-223 expression levels.

### Western blotting

Cells were lysed with ice-cold RIPA buffer (50 mmol/LTris-HCl, pH 7.5; 150 mmol/L NaCl; 1% NP-40; 0.25% sodium deoxycholate, 1 mmol/L EDTA), supplemented with a protease inhibitor cocktail (Sigma-Aldrich). Protein concentration was determined using a conventional Coomassie Bradford protein assay kit (Bio-Rad). Equal amounts of extracts (30 μg) were then electrophoresed on sodium dodecyl sulfate polyacrylamide gel electrophoresis gel (SDS-PAGE) and electroblotted to PVDF membranes (Bio-Rad). Membranes were immersed in blocking buffer (5% degreased milk powder) for 1 h and incubated with primary antibodies overnight at 4 °C. The primary antibodies used are as follows: rabbit polyclonal anti-RhoB, -MYPT1, -MLC2, -PCNA (1:1000 dilutions, Proteintech Group), rabbit polyclonal anti-MYPT1-Phospho (thr696) (1:500 dilutions, Cell Signaling), anti-MLC2-phospho (1:200 dilutions, Cell Signaling), beta-actin (Santa Cruz Biotechnology). They were then incubated with horseradish peroxidase-conjugated secondary antibodies (Jackson Immuno Research), and the protein bands were afterward visualized using the SuperSignal chemiluminescent detection module (Pierce) and exposed to X-ray film.

### 3′-UTR luciferase reporter assay

We applied TargetScan algorithm (http://www.targetscan.org) and FINDTAR3 (http://bio.sz.tsinghua.edu.cn/) to predict targets and the miRNA binding sites. The 3′-UTR of predicted target genes were PCR amplified and inserted into pmirGLO dual-luciferase vector (Promega). RhoB 3′-UTR were amplified from rat genomic DNA with primer 5′-GTC GAA TTC AGC AAG CCA CTA CTG TTG CTC CAT G-3′ (forward) and 5′-AGC TCT CGA GTC TTC TGA CAC TAT TAA GCC ACA GG-3′ (reverse). To construct mutated 3′-UTR report vector, two residues in the region that base-pairs with miRNA seeding sequences were mutated by site-directed mutagenesis. The pimers used were listed as follows: RhoB-mut1, 5′-CCC TGG GGA AGA CAT TTG CAT GAC TCT TGG GGT CGA GAG GAA GCA-3′(forward) and 5′-TGC TTC CTC TCG ACC CCA AGA GTC ATG CAA ATG TCT TCC CCA GGG-3′ (reverse); RhoB-mut2, 5′-GTG GTA CTT CTA AAT TGT CTT GTT TTG TTT TTG TTT TAT TTT TTT TAA ATA ATG ACT CAG ATG ACA AAT GGT GAA CTT ATG ATG-3′ (forward) and 5′-CAT CAT AAG TTC ACC ATT TGT CAT CTG AGT CAT TAT TTA AAA AAA ATA AAA CAA AAA CAA AAC AAG ACA ATT TAG AAG TAC CAC-3′ (reverse). The primers used for amplifying rat MLC2 3′-UTR and constructing its mutated reporter vector are listed as follows: MLC2 3′-UTR, 5′-GGA GAA TTC AGC CCC CTG ACA CCC CAG CCC CCG C-3′ (forward) and 5′- CCA CTC GAG AGA TTC TGC CCC CTC TGG GAC AGA T-3′ (reverse); MLC2-mut, 5′-AGA AGT GCG TGC CGA CGA CTC GCA GAT GTT CCC ACA-3′ (forward) and 5′-TGT GGG AAC ATC TGC GAG TCG TCG GCA CGC ACT TCT-3′ (reverse). HEK293 cells were seeded in 24-well plates and after the cells reached 80~90% confluence, each well of cells was transfected with 100 ng of 3′-UTR reporter vectors or mutated 3′-UTR reporter vectors, 900 ng of miR-223 expressing plasmid (pLVX-CMV-miR-223) with 2 μl of Lipofectamine 2000 (Invitrogen, Carlsbad, CA). The pLVX-CMV (Clontech) without miRNA sequences was used as the control. Two days after transfection, the cells were harvested for luciferase activity assay and measured independently with a Lumat LB9508 luminometer (Berthold, Bad Wildbad, Germany). Firefly luciferase activity was normalized to renilla luciferase activity.

### Rho GTPases Acthivity

Rho GTPases activity was determined by using Rho Activation Assay Biochem Kit (Cytoskeleton Inc., Denver, CO), and followed the procedures suggested by the manufacturer. Active RhoB were pulled down by rhotekin-RBD beads. Affinity-precipitated RhoB proteins were resolved by SDS-PAGE and detected by Western blot analysis using anti-RhoB antibody.

### Human Serum Collection

Human serum samples were collected from healthy participants and patients with congenital heart disease associated with pulmonary arterial hypertention (CHD-PAH) at the Sun Yat-Sen Cardiovascular Hospital (Shenzhen, China) and Fuwai Hospital (Beijing, China) in accordance with the guidelines approved by the Ethics Committee of Shenzhen University and the associated hospitals. The blood samples were allowed to stand at room temperature for 1 h and then centrifuged at 3,000 × g for 10 min at 4 °C. The supernatants were stored at −80 °C. The study was approved by both the ethics committees of the Sun Yat-Sen Cardiovascular Hospital and Fuwai Hospital. All subjects who participated in the study provided written informed consent.

### Statistical analysis

All data shown are mean values of at least three experiments, each performed in triplicate, with standard errors (SE). The differences between only two groups were analyzed using the double-sided Student’s *t* test, and with three or more groups were analyzed using ANOVA test. A *p* value less than 0.05 was considered significant. The significant differences (*p* < 0.05 or *p* < 0.01) are indicated in the figures by asterisk.

## Additional Information

**How to cite this article**: Zeng, Y. *et al.* MicroRNA-223 Attenuates Hypoxia-induced Vascular Remodeling by Targeting RhoB/MLC2 in Pulmonary Arterial Smooth Muscle Cells. *Sci. Rep.* **6**, 24900; doi: 10.1038/srep24900 (2016).

## Supplementary Material

Supplementary Information
