# Supplementary material for: MicroRNA-223 Attenuates Hypoxia-induced Vascular Remodeling by Targeting RhoB/MLC2 in Pulmonary Arterial Smooth Muscle Cells
Source: Sci Rep. 2016 Apr 28;6:24900. doi: 10.1038/srep24900 (PMC4848472; doi:10.1038/srep24900)
Supplement: Supplementary Information [file srep24900-s1.pdf]

MicroRNA-223 Attenuates Hypoxia-induced Vascular Remodeling by Targeting RhoB/MLC2 in Pulmonary Arterial Smooth Muscle Cells

Yan Zeng[1,2†], Xiaoying Zhang[1†], Kang Kang[3†], Jidong Chen[1], Zhiqin Wu[1], Jinyong Huang[1], Wenju Lu[4], Yuqin Chen[4], Jie Zhang[4], Zhiwei Wang[5], Yujia Zhai[5], Junle Qu[2], Ramaswamy Ramchandran[6], J. Usha Raj[6], Jian Wang[4,7*], Deming Gou[1*]

Supplemental Figures and Figure Legends

Supplemental Figure S1. Quantitative real-time PCR showing increased expression of miR-223 in rat and human PASMC transfected with miR-223 mimic (miR-223), compared with its scramble control (miR-Con). Data are shown as mean ± SE, ***p*<0.01.

Supplemental Figure S2. (A) Relative miR-223 levels measured by quantitative PCR in cells transfected with inhibitor against miR-223 (anti-223) and its scramble control (anti-Con). (B) EdU incorporation assay showing proliferation activity of miR-223 inhibited cells and its control cells under normoxia. The data above are shown as means ± SE, **p*<0.05 and ***p*<0.01 vs anti-Con. (C) Cell proliferation was determined by PCNA immunoblotting. β-actin was used as loading control.

Supplemental Figure S1.

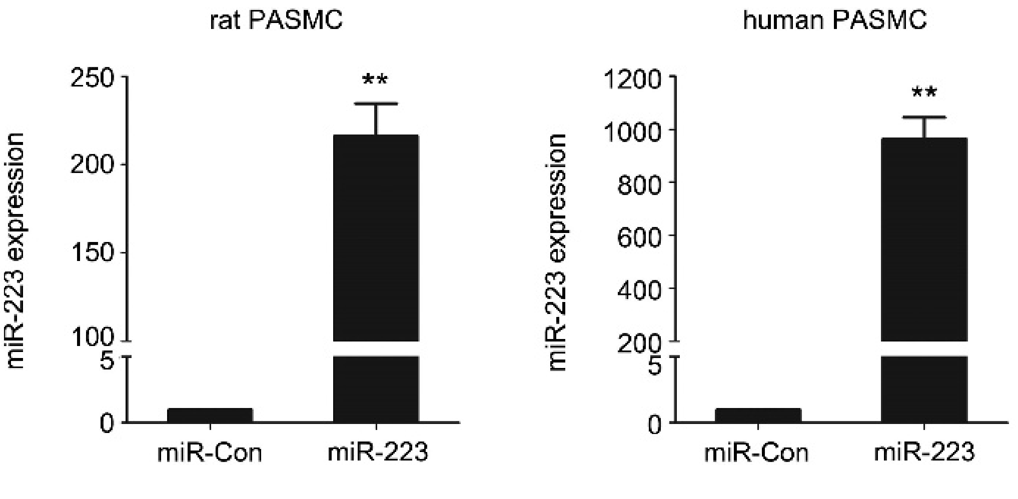

Supplemental Figure S2.

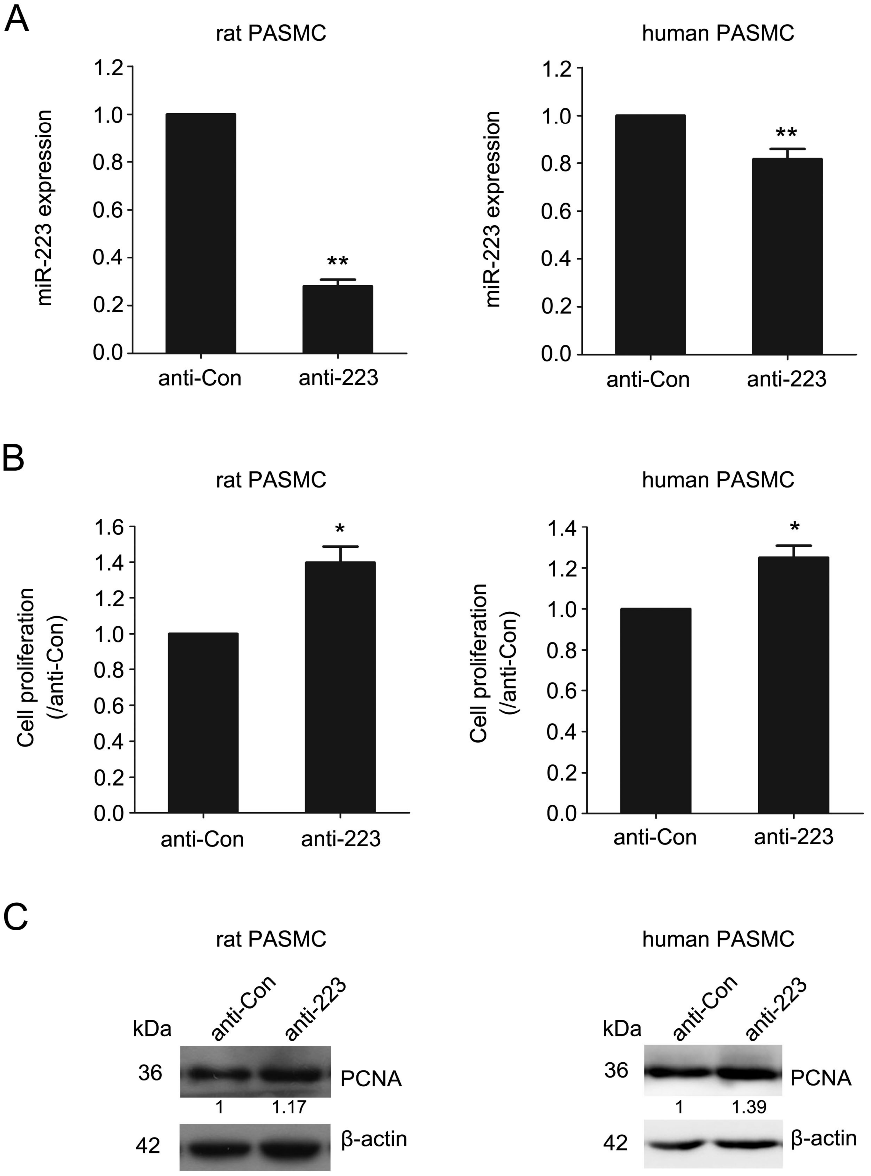

Supplemental Tables

Table S1. The information of the first batch of CHD-PAH patients and healthy donors

Sample ID	Gender	Age	Diagnosis	$2^{-\Delta Ct}$
1	Male	10	VSD,Preoperative, PH	0.154
2	Male	2	VSD, ASD, PH	2.827
3	Male	4	VSD, Preoperative	1.327
4	Male	0.5	VSD, Preoperative	0.402
5	Male	2	VSD, Preoperative	1.353
6	Male	4	CHD, VSD, Preoperative	0.552
7	Male	0.8	VSD, CHD, PFO	1.391
8	Male	0.5	VSD	0.252
9	Male	1	CHD、VSD	1.646
10	Male	37	ASD	0.400
11	Male	20	ASD, Preoperative	0.825
12	Male	20	ASD, Postoperative, Eisenmenger's syndrome	1.509
13	Male	28	ASD	0.144
14	Male	27	Healthy donor	0.407
15	Male	48	Healthy donor	0.660
16	Male	44	Healthy donor	0.856
17	Male	28	Healthy donor	0.274
18	Male	27	Healthy donor	0.499
19	Male	30	Healthy donor	0.263
20	Male	38	Healthy donor	1.499
21	Male	34	Healthy donor	0.771
22	Male	23	Healthy donor	1.027
23	Male	24	Healthy donor	0.577
24	Male	32	Healthy donor	1.125
25	Male	25	Healthy donor	1.888
26	Male	37	Healthy donor	0.339
27	Female	65	ASD, Severe PAH, Postoperative	0.336
28	Female	49	VSD, Preoperative	0.161
29	Female	39	VSD, Extremely Severe PAH	0.362
30	Female	22	ASD, Preoperative	0.394
31	Female	15	VSD, Preoperative	0.304
32	Female	28	VSD, Preoperative	0.468
33	Female	46	CHD, MVR, Postoperative	0.953
34	Female	3	VSD	1.834
35	Female	28	VSD, Severe PAH	0.161
36	Female	27	VSD, Severe PAH	0.430
37	Female	25	ASD	0.644
38	Female	20	VSD	1.190
39	Female	44	VSD, Preoperative	0.841

40	Female	27	VSD, Severe PAH	0.893
41	Female	39	VSD, Preoperative	0.514
42	Female	15	VSD, Preoperative	1.462
43	Female	19	ASD +VSD, Preoperative, Severe PAH	0.199
44	Female	26	Healthy donor	1.577
45	Female	36	Healthy donor	1.375
46	Female	39	Healthy donor	0.245
47	Female	43	Healthy donor	1.144
48	Female	27	Healthy donor	0.497
49	Female	28	Healthy donor	0.832
50	Female	30	Healthy donor	0.961
51	Female	28	Healthy donor	1.069
52	Female	32	Healthy donor	1.343
53	Female	27	Healthy donor	1.891
54	Female	26	Healthy donor	1.977
55	Female	44	Healthy donor	1.824
56	Female	22	Healthy donor	1.374
57	Female	35	Healthy donor	2.969
58	Female	27	Healthy donor	1.376
59	Female	27	Healthy donor	1.452
60	Female	33	Healthy donor	0.916

Notes:VSD, ventricular septal defect; ASD, atrial septal defect; CHD, congenital heart disease; PFO, Patent Foramen Ovale;MVR, mitral valve replacement.

Table S2. The information of the second batch of CHD-PAH patients and healthy donors

Sample ID	Gender	Age	Diagnosis	Remarks	$2^{-\Delta Ct}$
1	Male	35	ASD, Postoperative		2.431
2	Male	60	MS, Moderate PH		1.130
3	Male	42	VSD, Moderate-Severe		0.664
4	Male	32	VSD		2.190
5	Male	5	ASD		1.912
6	Male	37	ASD		1.334
7	Male	42	VSD, Moderate-Severe	Cardiac Catheterization,91/46 mmHg	0.755
8	Male	35	Healthy donor		1.498
9	Male	58	Healthy donor		1.155
10	Male	42	Healthy donor		2.430
11	Male	32	Healthy donor		3.086
12	Male	6	Healthy donor		2.528
13	Male	37	Healthy donor		5.457
14	Male	42	Healthy donor		2.013
15	Female	25	PDA, Severe	Cardiac Catheterization,105/68 mmHg	0.876
16	Female	35	IPAH, Severe	B-scan ultrasonography,106/56 mmHg	0.675
17	Female	27	ASD, Mild	B-scan ultrasonography, 34 mmHg	1.145
18	Female	29	ASD, Severe	B-scan ultrasonography, 93/160 mmHg	2.378
19	Female	24	ASD, Mild	B-scan ultrasonography, 34 mmHg	0.895
20	Female	18	ASD, Mild	B-scan ultrasonography, 37 mmHg	4.415
21	Female	20	VSD, Postoperative, Severe		2.969
22	Female	31	VSD, Severe		0.737
23	Female	33	ASD, Postoperative		1.135
24	Female	37	ASD, Moderate-Severe		6.193
25	Female	42	MSI	B-scan ultrasonography, 42 mmHg	1.328
26	Female	38	MSI	B-scan ultrasonography, 50 mmHg	3.823
27	Female	45	ASD	B-scan ultrasonography, 42 mmHg	1.438
28	Female	45	ASD	B-scan ultrasonography, 39 mmHg	1.982
29	Female	32	ASD	B-scan ultrasonography, 38 mmHg	0.943
30	Female	64	MSI	B-scan ultrasonography, 35 mmHg	1.770
31	Female	30	ASD, Moderate-Severe	B-scan ultrasonography, 66 mmHg	1.554
32	Female	25	Healthy donor		14.407
33	Female	35	Healthy donor		2.812
34	Female	27	Healthy donor		9.941
35	Female	29	Healthy donor		4.060
36	Female	24	Healthy donor		8.008
37	Female	16	Healthy donor		4.646
38	Female	21	Healthy donor		3.451
39	Female	31	Healthy donor		3.914
40	Female	33	Healthy donor		7.515

41	Female	37	Healthy donor		4.040
42	Female	43	Healthy donor		3.414
43	Female	38	Healthy donor		3.242
44	Female	45	Healthy donor		4.382
45	Female	45	Healthy donor		1.537
46	Female	30	Healthy donor		2.578
47	Female	63	Healthy donor		3.754
48	Female	30	Healthy donor		5.457

Notes:ASD, atrial septal defect; MS, mitral stenosis; VSD, ventricular septal defect; PDA, Patent Ductus Arteriosus; IPAH, Idiopathic Pulmonary Arterial Hypertension; MSI, mitral stenosisand insufficiency.
